# Protection Before Impact: the Potential Neuroprotective Role of Nutritional Supplementation in Sports-Related Head Trauma

**DOI:** 10.1007/s40279-017-0847-3

**Published:** 2018-01-24

**Authors:** Jonathan M. Oliver, Anthony J. Anzalone, Stephanie M. Turner

**Affiliations:** Sports Concussion Research Group, Department of Kinesiology, Texas Christian University (TCU), Box 297730, Fort Worth, TX 76129 USA

## Abstract

Even in the presence of underreporting, sports-related concussions/mild traumatic brain injuries (mTBI) are on the rise. In the absence of proper diagnosis, an athlete may return to play prior to full recovery, increasing the risk of second-impact syndrome or protracted symptoms. Recent evidence has demonstrated that sub-concussive impacts, those sustained routinely in practice and competition, result in a quantifiable pathophysiological response and the accumulation of both concussive and sub-concussive impacts sustained over a lifetime of sports participation may lead to long-term neurological impairments and an increased risk of developing neurodegenerative diseases. The pathophysiological, neurometabolic, and neurochemical cascade that initiates subsequent to the injury is complex and involves multiple mechanisms. While pharmaceutical treatments may target one mechanism, specific nutrients and nutraceuticals have been discovered to impact several pathways, presenting a broader approach. Several studies have demonstrated the neuroprotective effect of nutritional supplementation in the treatment of mTBI. However, given that many concussions go unreported and sub-concussive impacts result in a pathophysiological response that, too, may contribute to long-term brain health, protection prior to impact is warranted. This review discusses the current literature regarding the role of nutritional supplements that, when provided before mTBI and traumatic brain injury, may provide neurological protection.

## Introduction

It is estimated that between 1.6 and 3.8 million sports-related concussions occur per year in the United States (US) [1, 2]. A more recent report estimates that a majority of those injuries (1.1–1.9 million) occur in those younger than 18 years of age [3]. This statistic is reflected by an increase in the number of sports-related concussions reporting to the emergency department in the US [4]. Similar patterns of increase have been reported internationally [5–7]. However, those estimates may not represent actual statistics as an estimated 53% of those who have a concussion do not report to appropriate medical personnel [8]. Often used interchangeably with mild traumatic brain injury (mTBI), concussion is a type of mTBI resulting from either direct or indirect impact to the head. Described by the 5th International Conference on Concussion in Sport as, “a traumatic brain injury induced by biomechanical forces” [9], a concussion is largely an injury that manifests as short-term impairment in neurological function. The overt damage associated with more severe traumatic brain injury (TBI) and often detectable via traditional radiography is not characteristic of a sports-related concussion [9–11]. The seriousness of concussion in sport is highlighted by the fact that athletes with a previous concussion are at a greater risk of future concussions [12] and repetitive concussions may increase the likelihood of developing long-term neurological complications [13]. Further, young athletes are at an increased risk for concussions compared with collegiate and professional athletes, and recover at a slower rate [2, 9, 14]. Those disparities are attributed to poor tackling techniques, insufficient medical staff, and incomplete brain development [15].

Whether the increase in sports-related concussions is due to greater awareness or as a result of increased sport participation is not known. Organized team sports account for nearly 75% of sports-related concussions among children 8–19 years old [1]. The National Collegiate Athletic Association (NCAA) Sports Sponsorship and Participation Rates Report states the number of athletes participating in NCAA sports has increased substantially over the last several decades [16], which is representative of growth at lower levels of competition [17]. American Football boasts the highest participation rates in the US [17], with soccer holding the primary spot internationally and exhibiting patterns of continued growth. Among contact sports, American Football is associated with the highest incidence of sports-related concussion, followed by soccer, basketball, and wrestling [18]. However, to focus solely on sports-related concussion ignores the growing body of evidence that sub-concussive impact associated with sport participation may also have important implications for long-term brain health [19]. With advancing technologies, the ability to detect damage associated with sub-concussive impacts is emerging. A multitude of studies have demonstrated that sub-concussive impacts, in the absence of a concussion diagnosis, result in quantifiable pathophysiological changes as demonstrated via advanced imaging [20–25] and fluid biomarker quantification [26–30]. Further, younger athletes [24, 31] and those participating in contact sports with less likelihood for contact (i.e., soccer) [32, 33] are not immune to damage resulting from sub-concussive impacts. Head trauma associated with a lifetime of sports participation may lead to neurological impairments and debilitating neurodegenerative disorders [34, 35]. Chronic traumatic encephalopathy (CTE), a debilitating neurodegenerative disease that has long been associated with sport participation, may indeed be linked to repetitive concussive and sub-concussive impacts [36]. In fact, a recent investigation that conducted postmortem analysis of the brain tissue from 85 individuals with a history of repetitive mTBI, 80 of whom were athletes, reported that 68/85 (80%) displayed pathology of CTE [35]. However, as most of the available evidence is derived from postmortem investigations, there are many yet-to-be-rectified methodological limitations [37] that leave the field with many unanswered questions regarding the definitive impact of repetitive sub-concussive and concussive head trauma on the development of CTE, which must be addressed.

The American Medical Society for Sports Medicine position statement for concussion in sport recently suggested that protective equipment does not reduce the incidence and/or severity of concussion in sport [38], despite helmet advancements for example [39]. This highlights the need for different approaches to prevent and/or reduce the deleterious effects of sports-related concussion and sub-concussive impacts. The current consensus regarding sports-related concussion management and treatment recommends physical and cognitive rest until the resolution of acute symptoms followed by a period of graduated exertion until the athlete can participate without symptom exacerbation [9]. The conservative nature of the ‘rest and wait’ style of treatment is not only safe but succeeds in avoiding exacerbation of symptoms. However, simply allowing for spontaneous recovery to occur may not be optimal and is not commonplace amongst the management of other injuries or diseases. In fact, recent reports highlight the use of cervical and vestibular rehabilitations as well as submaximal exercise as treatment strategies for concussion and post-concussion syndrome [40]. Pharmacological therapy is recommended for the management of only a limited number of protracted concussion symptoms such as sleep disturbances and mood disorders. However, there is no evidence to date that pharmacological therapy is effective for attenuating recovery duration or providing neuroprotection before impact. Most clinical trials attempting to treat TBI have failed to report any benefits of targeted drug treatment [41, 42]. Perhaps more importantly, treatment does not account for those injuries that go unreported or the sub-concussive impacts that also result in a pathophysiological response. Outside of changes to rules and regulations, which have been done, there is no consideration for mitigating the deleterious effects of sub-concussive or concussive impacts. As the field has developed an enhanced understanding of the pathophysiology of concussion, pre-clinical studies that mutually target several pathophysiological consequences of the injury cascade resulting from concussive and sub-concussive impact are suggested. Nutritional supplementation and nutraceuticals have shown promise in this area when provided before and after mTBI and TBI. In order to adequately review the current available literature, the focus of this review is to discuss the literature related to nutritional supplementation provided before mTBI and TBI occur to determine if a potential neuroprotective role exists. Before discussing those specific interventions, a brief summary of the underlying pathophysiology is warranted.

## Pathophysiology

It is important to note that our understanding of the pathophysiological consequences attributed to concussion have been gleaned from animal models, the majority being rodent. Unfortunately, there are also inherent difficulties in reproducing the biomechanical forces that result in mTBI and more specifically those that are responsible for sports-related concussions, as the primary difference is the heterogeneity of those forces that occur in sports (reality) compared with the homogeneity of the models developed in rodents. For a more extensive review of the gap between the laboratory and reality, the reader is referred to a recent review by Angoa-Pérez et al. [43]. Despite differences, the animal models of mTBI and TBI have provided a wealth of information on the pathophysiological response discussed herein.

It is well established that concussion is a multifaceted condition that affects both the structural and metabolic organization of the brain [44, 45]. At a macro level, overt hemorrhage or edema are not present as the majority of computed tomography (CT) scans exhibit no gross pathology. Instead, concussion manifests as functional disturbance within the brain [46, 47]. Though no gross anatomical changes are present, the symptoms associated with concussion are indeed a result of pathophysiological events at the cellular level.

Initially, the rotational and linear forces that characterize the insult induce vulnerability to the structural integrity of the neuronal plasma membrane. This primary event allows for a dysregulation of ionic flux such that there is an efflux of potassium (K^+^) and a massive influx of both sodium (Na^+^) and calcium (Ca2^+^). As the shift in membrane potential is observed there is subsequent uninhibited release of excitatory neurotransmitters, primarily glutamate [44, 45, 47–49]. Localized depolarization propagates down the neuron via *N*-methyl-d-aspartate (NMDA) receptors and spreads to neighboring neurons to create a diffuse cellular “depression-like” state [44, 45, 47–49]. In order to restore resting membrane potential and ionic concentration gradients, large amounts of adenosine triphosphate (ATP) are required to power Na^+^/K^+^ ATPase pumps. As a result, there is an upregulation of glycolytic pathways [34, 44, 45]. However, at the same time, decreased cerebral blood flow reduces delivery of glucose to the cells with increased need, thus creating a mismatch between supply and demand for glucose within the brain [34, 44, 45, 47, 50]. Furthermore, the influx of calcium, a potent second messenger capable of activating apoptotic pathways, is sequestered into the mitochondria, contributing to eventual mitochondrial dysfunction, further impairing the ability of the cell to produce glucose through oxidative phosphorylation [44, 45, 49, 51]. Reliance upon anaerobic oxidative pathways to meet the ATP demands on the cell results in an acute accumulation of lactate and local acidosis [44, 45, 48]. While lactate can be a fuel source for neurons to help meet ATP demands, this process requires proper mitochondrial function and aerobic pathways [44]. Following acute hyperglycolysis, injured neurons enter a state of hypometabolism, possibly lasting until complete resolution of symptoms [45]. Lastly, the ionic imbalances can alter the redox state of the cell allowing for the production of reactive oxygen species (ROS) [45, 47]. The production of ROS and the resultant oxidative stress reduces the antioxidant defenses of the cell allowing cellular damage, primarily lipid peroxidation [47].

As a potent second messenger, calcium is also capable of inducing conformational changes in axonal proteins. Phosphorylation of neurofilaments or calpain-mediated proteolysis can result in compaction, reduced integrity of the axon, and impaired axonal transport [44, 45, 49, 52]. Further cytoskeletal damage occurs to microtubules as a direct result of the mechanical forces on the neurons [44, 45, 48]. Consequent to this, there is a backup of transport products and axonal swelling is evident [44, 45, 48, 49]. A result of microtubule breakdown is the release of microtubule-associated protein tau, a protein involved in assembly and stabilization of microtubules [48]. However, breakdown of microtubules as well as phosphorylation at serine or threonine residues of tau can result in decreased binding affinity of tau to microtubules and increased affinity for self-binding [48]. Progression of tau pathology results in helical formation of tau subunits, ultimately resulting in neurofibrillary tangles (NFTs), one of the defining pathologies of CTE [34].

Like most injuries, an inflammatory response also develops following head injury in order to activate the immune system [45, 46, 53, 54]. Controlled cortical impact (CCI) in rats revealed a biphasic inflammatory response, with chemokine C-X-C motif ligand 1 (CXCL1), interferon-γ (IFN-γ), interleukin (IL)-13, tumor necrosis factor-α (TNF-α), and IL-4 peaking within 4 h of injury, while chemokine C–C motif ligand (CCL)-2, CCL20, and IL-1β did not reach peak concentrations until 12 or 24 h post-injury and remained elevated for as long as 7 days. IL-5 showed no elevation in response to CCI. Of the tested cytokines, IL-13 and IL-4 were the only ones to exemplify anti-inflammatory properties through downregulating cytokine expression and altering activation of microglia cells [53].

Other animal studies have focused on gene expression instead of protein production [54]. High throughput screening and microarray analysis revealed altered expression in 23 genes following fluid percussion injury (FPI), including genes implicated in both inflammatory and apoptotic pathways. Evaluation of temporal profiles revealed that the inflammatory response (at ~ 3 h) preceded the apoptotic response (6–48 h) [54]. Interestingly, the gene expression and clear histopathological signs of apoptosis revealed no apparent tissue loss following injury [54], confirming TBI represents a functional disturbance rather than structural disturbance to the brain [9, 44–47, 55, 56]. Although apoptosis, defined as programmed cell death, produces no inflammatory response in adjacent cells, the time-dependent profile delineated here suggests the production of pro-inflammatory cytokines such as IL-1β and TNF-α by the innate immune system results in activation of this programmed pathway [54].

The post-concussive inflammatory response and cytokine release is a neuroprotective mechanism to initiate repair processes immediately following injury [56]. However, prolonged activation of this pro-inflammatory state can become neurotoxic, having detrimental effects on brain health such as development of neurodegenerative diseases [56].

## Nutritional Supplementation

As discussed in the previous section, the pathophysiology resulting from a sports-related concussion is complex and multifaceted as evidenced from animal models of mTBI and TBI. While pharmaceutical therapies may target only one mechanism of injury and have not shown great promise, nutritional supplementation has emerged as a potential neuroprotective agent that targets multiple mechanisms within the complex secondary sequelae. By targeting multiple mechanisms within the injury cascade, nutritional supplementation may be used prior to injury to allay damage that occurs subsequent to both sub-concussive and concussive incidents. In the following sections, nutritional supplementation as a neuroprotective agent, provided prior to injury, will be examined.

### Creatine

In the days following sports-related concussion, proton magnetic resonance spectroscopy has detected reduced brain levels of creatine (Cr) [N-aminoiminomethyl-*N*-methylglycine] coincident with decreases in *N*-acetylaspartate (NAA) [57]. Changes in NAA have been shown to correlate with those of adenine nucleotides, validating the use of NAA as a surrogate marker of cerebral energy metabolism during the vulnerable period following mTBI [44, 58, 59]. In the brain and other tissues with high fluctuating energy demands, Cr serves to maintain temporal and spatial energy homeostasis in conjunction with the creatine kinase/phosphocreatine (CK/PCr) system [60–64]. The tight coupling of Cr to the CK/PCr system and the dysregulation of energy metabolism that follows injury explains why reduced brain levels of Cr are observed in the period following an mTBI [57, 65, 66]. However, several studies have reported no changes in Cr in the presence of decreased NAA [67, 68]. A closer examination of that study in which brain levels of Cr were reduced suggests that the reduction in Cr resulted from a more severe injury, as athletes in that study experienced a longer duration of clinical symptoms post-injury, as well as a longer period of time for the normalization for NAA [57]. Therefore, if brain Cr levels were to be made available by increasing the pool of brain Cr prior to mTBI, theoretically, the increased availability may provide an additional energy source allowing a maintenance of energy homeostasis during periods of energy fluctuations. Indeed, two reports in rodent models of TBI (controlled cortical impact) have concluded that Cr supplementation prior to insult afforded those supplemented animals neuroprotection through direct action on mitochondrial energy homeostasis [69, 70]. Cr-supplemented animals exhibited reduced free fatty acid and lactate accumulation as well as a reduction in reactive oxygen intermediate production. The apparent maintenance of energy homeostasis and resultant decrease in oxidative stress likely contributed to the cortical tissue sparing that was also observed in those animal supplemented with Cr. A reduction in oxidative stress reduces subsequent lipid peroxidation which can further damage neuronal proteins [71–73]. The potential neuroprotective effect of Cr may not be limited to energy homeostasis as recent reports suggested additional roles for Cr within the brain including, but not limited to, action on NMDA [74] and gamma-aminobutyric acid (GABA) receptors [75]. Further study is warranted in models of mTBI and TBI.

Cr is endogenously synthesized from glycine, arginine, and S-adenosyl-l-methionine in the kidneys, liver, pancreas [63], and to a lesser extent, the brain [76]. Cr synthesized in the brain [76] does not account for the total pool of brain Cr as Cr is also shuttled across membranes via a creatine transporter protein (CrT) [55, 77, 78]. Since the CrT is not ubiquitously expressed in the brain, Cr uptake and saturation of the endogenous pool takes longer than in other tissues, specifically muscle [55, 79], the primary site of Cr storage accounting for up to 90% of the total body Cr pool [63, 80]. This likely explains why animals supplemented for a longer period and at higher doses were afforded greater protection [69, 70] and highlights the importance of timing and dosage as factors contributing to neuroprotection (Table 1). The total body Cr pool can be increased by ingestion of foods high in Cr (i.e., meat or fish) and/or nutritional supplementation. Ingestion of Cr in solution may increase whole-body Cr to a greater degree than meat [81], which is affected by cooking processes [82]. One consideration for athletes seeking to increase Cr levels is the additional calories associated with increased consumption of meat and/or fish. The most common form of Cr found in dietary supplements, food products, and referred to in the scientific literature is creatine monohydrate (CrM) [83]. Though the authors are unaware of any study examining the efficacy of other forms of Cr on the pool of Cr in the brain, a number of studies have been carried out on the effects of various formulations on muscle Cr. Despite consistent findings demonstrating the efficacy of CrM for increases in muscle Cr [83–85], manufacturers continue to develop alternative forms in an effort to increase market share.

**Table 1:** Outline of studies in which creatine or curcumin was provided prior to injury, mild traumatic brain injury, or traumatic brain injury

Study	Animal injury model	Supplementation dosing	Pathophysiological outcomes	Functional outcomes
Sullivan et al. [70]	Sprague–Dawley rats Controlled cortical impact	RD CrD 4 weeks before injury	CrD-fed rats had significantly less cortical tissue damage than RD-fed rats CrD-fed rats had significantly higher mitochondrial membrane potential than RD-fed rats CrD-fed rats had significantly fewer ROI than RD-fed rats CrD-fed rats had significantly less intramitochondrial Ca^2+^ than RD rats	
Sullivan et al. [70]	ICR mice Controlled cortical impact	Intraperitoneal olive oil (0.1 mL·10 g_BW_^−1^ day^−1^) + Cr (3 mg kg^−^ day^−1^) 1, 3, or 5 days prior to injury	Cr supplementation for 3 and 5 days prior to injury exhibited significantly less cortical tissue damage than Cr supplementation 1 day prior to injury and no creatine supplementation	
Scheff and Dhillon [69]	Sprague–Dawley rats Controlled cortical impact	RD CrD 0.5% CrD 1.0% 2 weeks prior to injury	CrD-fed rats had significantly less cortical tissue damage than RD-fed rats but there was no significant difference between 0.5% Cr and 1% Cr	
Wu et al. [73]	Sprague–Dawley rats Mild fluid percussion injury	RD (13% energy from fat) HF (39% energy from fat) RD + 500 ppm curcumin HF + 500 ppm curcumin	Curcumin-fed rats had less post-TBI oxidative damage than RD-fed rats Curcumin-fed rats with normalized post-TBI levels of hippocampal BDNF, synapsin I, and CREB than control-fed rats	Curcumin-fed rats performed better in post-TBI Morris water maze testing compared with RD fed rats
Laird et al. [101]	CD-1 mice Controlled cortical impact	Intraperitoneal curcumin 75, 150, or 300 mg kg^−1^ 15 min prior to TBI, 30 min post-TBI, or 60 min post-TBI	Pretreatment with 75 or 150 mg kg^−1^ curcumin significantly reduced brain water content Pretreatment with 150 mg kg^−1^ curcumin significantly reduced expression of AQP4 Pretreatment with 150 mg kg^−1^ curcumin significantly reduced IL-1β expression Pretreatment with 150 mg kg^−1^ curcumin significantly reduced NF-κB expression	Pretreatment with 150 mg kg^−1^ curcumin significantly improved overall locomotion and movement within squares in the center of the open-field chamber after TBI Motor performance was unaffected by pretreatment with curcumin
Samini et al. [72]	Wistar rats Controlled cortical impact	Intraperitoneal curcumin 50 mg kg^−1^ day^−1^ 100 mg kg^−1^ day^−1^ 5 days before injury	Pretreatment with 100 mg kg^−1^ curcumin significantly reduced the size of brain lesions Pretreatment with 100 mg kg^−1^ curcumin significantly diminished post-TBI lipid peroxidation	Pretreatment with curcumin significantly improved sensory-motor performance
Sharma et al. [102]	Sprague–Dawley rats Mild fluid percussion injury	RD Curcumin diet Standard rat chow + 500 ppm curcumin	Curcumin-fed rats with normalized post-TBI levels of hippocampal pAMPK/AMPK ratio, uMtCK, UCP2, COX-II, and Sir2 compared with RD-fed rats	

*AMPK* AMP-activated protein kinase, *AQP4* aquaporin 4, *BDNF* brain-derived neurotrophic factor, *COX*-*II* cytochrome c oxidase II, *Cr* creatine, *CrD* regular diet enriched with 1% creatine monohydrate, *CREB* cyclic adenosine monophosphate response element-binding protein, *HF* high-fat diet, *ICR* Institute of Cancer Research, *IL*-*1β* interleukin-1β, *NF*-*κB* nuclear factor-kappa B, *p*-*AMPK* phosphorylated AMP-activated protein kinase, *ppm* parts-per million, *RD* regular diet, *Sir2* silent information regulator 2, *TBI* traumatic brain injury, *UCP2* mitochondrial uncoupling protein 2, *uMtCK* ubiquitous mitochondrial creatine kinase

CrM supplementation is an effective strategy for increasing levels of brain Cr in humans. A single bolus dose of CrM (20 g) has been reported to increase brain levels of Cr which are further enhanced when supplementation is extended [86]. However, a recent study found no difference in brain Cr content following a 7-day period in young (10–12 years), healthy participants [87]. In tissues with a high pre-supplementation pool of Cr, such as muscle and the brain, a period of 2–4 weeks is recommended [79]. A high pre-supplementation pool may limit any further increases. Further, in muscle, one of the most studied tissues related to Cr uptake, it is well known that some individuals respond to Cr supplementation (responders), while others do not (non-responders). This has been suggested to be due to the physiological profile of the individual (i.e., those with high initial levels may not respond) [84, 88].

A known and valid side effect of creatine supplementation is weight gain [89] and athletes who use creatine should be cautious, particularly if competing in a sport that is weight restricted or classified. Several case reports and an array of anecdotal evidence have misleadingly purported that oral creatine supplementation increases the risk of musculoskeletal cramping and causes dehydration and renal injury. However, there is no persuasive evidence suggesting that oral creatine supplementation causes musculoskeletal cramping or adversely affects renal function in healthy or clinical populations [89]. In fact, a 2003 study found that the incidence of muscle cramping, dehydration, and total injuries over the course of a collegiate American Football season was less in those athletes who supplemented with creatine when compared with those athletes not supplementing with creatine [90]. Furthermore, administration of creatine doses of up to 0.8 g/kg/day for up to 5 years have demonstrated no adverse health risks [89].

### Curcumin

In the presence of ROS, the high content of polyunsaturated fatty acids (PUFAs) make the brain particularly susceptible to lipid peroxidation [71], which can further damage neuronal proteins [72, 73, 91]. Curcumin, the bioactive component of the spice herb turmeric (*Curcuma longa*), has a long history of medicinal use due to its antioxidant and anti-inflammatory properties [92]. Several studies have demonstrated the antioxidant properties of curcumin in rodent models of mTBI [72, 73, 93]. Perhaps the most convincing argument for the antioxidative effects of curcumin following mTBI were demonstrated in the presence of a diet high in saturated fat [73]. Diets high in saturated fat increase free radical formation and exacerbate the deleterious effect of TBI on cognition and neuroplasticity [94]. Curcumin attenuated the increase observed in oxidized proteins and normalized levels of brain-derived neurotrophic factor (BDNF), synapsin I, and cyclic adenosine monophosphate response element-binding protein (CREB) similarly in those fed normal and high-fat diets [73]. BDNF and its downstream effectors synapsin I and CREB are pivotal for facilitation of synaptic transmission and modulation of transcription factors associated with cognitive processes [94–96]. The lack of cognitive deficits observed in those supplemented with curcumin on either diet in the presence of normal BDNF supports previous findings [94, 96].

Curcumin is also known for its anti-inflammatory properties. In that regard, curcumin interacts with multiple inflammatory pathways [97, 98], but it primarily suppresses inflammation by inhibiting IκB kinase (IKK) signaling complex, thereby preventing the activation of nuclear factor-kappa B (NF-κB) [97, 99, 100], which regulates the release of many pro-inflammatory cytokines, including IL-1β. IL-1β has been suggested to play a role in cerebral edema following TBI by way of aquaporin 4 (AQP4) regulation [101]. Pre-treatment with curcumin prior to injury attenuated cerebral edema concomitant with reduced NF-κB activation, IL-1β, and AQP4 expression (Table 1). However, recent findings point to the potential of curcumin to maintain energy homeostasis [102]. Though maintenance of energy homeostasis does not necessarily mean a complete amelioration of oxidative stress and inflammatory processes implicated in the pathological secondary sequelae, it would at least attenuate those secondary injury processes. Sharma et al. [102] reported that curcumin supplementation prior to injury effectively maintained energy homeostasis post-injury as evidenced by an increase in mitochondrial proteins, including AMP-activated protein kinase (AMPK) and ubiquitous mitochondrial creatine kinase (uMtCK). AMPK is an important sensor of cellular energy homeostasis [103]. Normalization of AMPK and p-AMPK following TBI is an indication of maintenance of cellular energy homeostasis. Excessive intracellular calcium is largely responsible for mitochondrial dysfunction and an increase in oxidative stress [104]. uMtCK is an enzyme responsible for the regulation of calcium and energy homeostasis [105], and the apparent preservation of energy homeostasis as evidenced by conservation of uMtCK, AMPK, and other mitochondrial proteins may therefore be responsible for the reduction in oxidative stress observed by others [72, 73, 93]. Though the exact mechanism by which curcumin may exert effects on energy regulation is unknown, it may be through modulation of the AMPK/uncoupling protein 2 (UCP-2) pathway [106–108].

Though the effects of curcumin appear to be dose dependent, with those fed higher doses afforded greater protection [72], curcumin is not highly bioavailable. A major limitation to the therapeutic potential of curcumin is the poor solubility, low absorption from the gut, rapid metabolism, and rapid systemic elimination [109]. Curcumin is primarily excreted through the feces, never reaching detectable levels in the circulation [110]. High doses of orally administered curcumin, upwards of 10–12 g, have been reported to result in little to no appearance in the circulation [111]. Various methods have been developed to increase the bioavailability of curcumin involving emulsions, nano-crystals, and liposomes with varying degrees of success [112]. As an example, a recent formulation of curcumin in combination with cellulosic derivatives and natural antioxidants (tocopherol and ascorbyl palmitate) was reported to result in a 46-fold increased absorption over a standardized curcumin mixture and improved absorption over other formulations (5.8- to 35-fold), and was well tolerated with no adverse effects reported [113]. Thus, improved bioavailability is possible and a number of unique formulations have already earned the distinction of being generally recognized as safe (GRAS) from the US Food and Drug Administration.

### Omega (ω)-3 Fatty Acids

A unique pathological consequence of TBI is that there is a reduction in the quantity of neuronal docosahexaenoic acid (DHA) following injury [93, 114]. Further, deficiency of brain DHA content (70%), induced by dietary restriction, heightens the response to TBI (cortical controlled impact in rats) as evidenced by greater breakdown of neuronal cytoskeleton protein (alpha spectrin II), exacerbated cell death (fewer NeuN positive cells), slower recovery of motor function, more anxiety-like behaviors, and cognitive deficits [115, 116]. DHA, an ω-3 long-chain PUFA (LCPUFA), is present in a variety of tissue types, but is most highly concentrated within the mammalian central nervous system (CNS) and is over 100-fold more abundant within the mammalian CNS than eicosapentaenoic acid (EPA), another ω-3 LCPUFA [117]. As such, the study of ω-3 fatty acids (FAs), specifically DHA intake and supplementation as it pertains to neurological development, disease, and functionality, has been extensively investigated [118–121]. Despite the fact that EPA is not an integral constituent of the CNS [119, 120], many studies examining the effects of ω-3 FAs on brain function have supplemented subjects with fish oil, which comprises both EPA and DHA. For a more extensive appraisal regarding the role of ω-3 FAs and brain health, refer to the following reviews [122–125].

In animal models of mTBI and TBI, prophylactic supplementation with ω-3 FAs, specifically DHA, mitigates white matter damage, a characteristic of mTBI, as evidenced by fewer β-amyloid precursor-positive axons; enhanced preservation of myelin; and enhanced protection of neurofilament morphology [126–128] (Table 2). The mechanism by which a neuroprotective effect is realized is multifaceted and not completely understood. A series of mutual mechanisms by which DHA may convey neuroprotective effects are those characteristic of the complex pathological sequelae that occurs post-injury. DHA has been shown to allay glutamate cytotoxicity [129, 130], suppress mitochondrial dysfunction and the eventual development oxidative stress [131], decrease calcium influx [130], and downregulate α-amino-3-hydroxy-5-methyl-4-isoxazolepropionic acid (AMPA) receptor subunits [132]. Animals supplemented with ω-3 FAs consistently exhibit enhanced resilience to TBI with functional outcomes mirroring those biological indicators of injury, even following multiple mTBIs [127], similar to that which would be observed in repetitive sports-related concussive injuries over a lifetime of play. While the obvious preservation of white matter undoubtedly aids in maintaining neurocognitive function following injury [126, 127, 133], the blunting of injury-induced reductions in molecular elements important for learning, including BDNF, synapsin I, and CREB also plays a role [133].

**Table 2:** Outline of studies in which ω-3 fatty acids or ω-3 fatty acids plus curcumin were provided prior to injury, mild traumatic brain injury, or traumatic brain injury

Study	Animal injury model	Supplementation dosing	Pathophysiological outcomes	Functional outcomes
Wu et al. [133]	Sprague–Dawley rats Mild fluid percussion injury	RD (0.9% DHA/1.0% EPA) FO (12.4% DHA/13.5% EPA) 4 wk prior to injury	FO-fed rats had less post-TBI oxidative damage than RD-fed rats FO-fed rats exhibited normalized post-TBI levels of hippocampal BDNF, synapsin I, and CREB compared with RD-fed rats	FO-fed rats performed better in post-TBI Morris water maze testing compared with RD-fed rats
Wu et al. [131]	Sprague–Dawley rats Mild fluid percussion injury	RD (0.9% DHA/1.0% EPA) FO (12.4% DHA/13.5% EPA) 4 weeks prior to injury	FO-fed rats had less post-TBI oxidative damage compared with RD-fed rats FO-fed rats exhibited normalized post-TBI levels of hippocampal Sir2α, AMPK, p-AMPK, and uMtCK compared with RD-fed rats	
Mills et al. [126]	Sprague–Dawley rats Marmarou impact acceleration injury	DHA only 0 mg kg^−^ day^−1^ 4 mg kg^−1^ day^−1^ 12 mg kg^−1^ day^−1^ 40 mg kg^−1^ day^−1^ 30 days prior to injury	40 mg kg^−1^ day^−1^ DHA-fed rats with 4.7 × the amount of serum DHA than lower doses 40 mg kg^−1^ day^−1^ DHA-fed rats with significantly fewer APP-positive axons than lower doses 40 mg kg^−1^ day^−1^ DHA-fed rats with significantly fewer caspase-3 positive axons than lower doses 40 mg kg^−1^ day^−1^ DHA-fed rats with significantly fewer CD-68 positive macrophages than lower doses	40 mg kg^−1^ day^−1^ DHA-fed rats performed better in post-TBI Morris water maze testing compared with lower doses
Pu et al. [127]	Male C57BL/6J mice Controlled cortical impact	RD (0.5% omega-3 fatty acids) FO (15 g kg^−1^ day^−1^ omega-3 fatty acids) 60 days prior injury	No significant difference in volume of cortical lesions No significant difference in the number of viable neurons within CA1 region of hippocampus FO-fed mice had significantly more viable neurons within CA3 region of hippocampus post-TBI compared with RD-fed mice FO-fed mice with significantly fewer post-TBI activated microglia and significantly less mRNA levels of IL-1β, TNFα, COX-II, and iNOS compared with RD-fed mice	FO-fed mice had fewer sensorimotor deficits compared with RD-fed mice FO-fed mice performed better in post-TBI Morris water maze testing compared with RD-fed rats
Wang et al. [128]	Sprague–Dawley rats Repetitive (2) mild fluid percussion injury	RD (0% fish oil) FO (0.6% DHA/0.8–1.0% EPA) 4 wk prior to injury	No significant difference in hippocampal neuron density between groups	FO-fed rats performed better in post-TBI Morris water maze testing compared with RD-fed rats
Wu et al. [73]	Sprague–Dawley rats Mild fluid percussion injury	RD Curcumin diet 500 ppm curcumin DHA diet (1.2% DHA) DHA + curcumin diet (500 ppm curcumin + 1.2% DHA)	Curcumin diet, DHA diet, and DHA + curcumin diet-fed rats normalized post-TBI levels of hippocampal BDNF and p-TrkB compared with RD-fed rats Curcumin diet, DHA diet, and DHA + curcumin diet-fed rats significantly reduced post-TBI lipid peroxidation compared with RD-fed rats Curcumin diet, DHA diet, and DHA + curcumin diet-fed rats normalized post-TBI levels of FADS2 and 17β-HSD4 compared with RD-fed rats Curcumin diet, DHA diet, and DHA + curcumin diet-fed rats exhibited preserved brain DHA content post-TBI compared with RD-fed rats	Curcumin diet, DHA diet, and DHA + curcumin diet-fed rats performed better in post-TBI Barnes maze testing compared with RD-fed rats

*AMPK* AMP-activated protein kinase, *APP*-*positive* amyloid precursor protein, *BDNF* brain-derived neurotrophic factor, *COX*-*II* cytochrome c oxidase II, *CREB* cAMP response element-binding protein, *DHA* docosahexaenoic acid, *EPA* eicosapentaenoic acid, *FADS2* Δ6 fatty acid desaturase, *FO* fish-oil diet, *IL*-*1β* interleukin-1β, *iNOS* inducible nitric oxide, *p*-*AMPK* phosphorylated AMP-activated protein kinase, *ppm* parts-per million, *p*-*TrkB* phospho-tropomyosin receptor kinase B, *RD* regular diet, *Sir2α* silent information regulator 2α, *TBI* traumatic brain injury, *TNFα* tumor necrosis factor alpha, *uMtCK* ubiquitous mitochondrial creatine kinase, *17β*-*HSD4* 17β-hydroxysteroid dehydrogenase type 4

Worldwide consumption of ω-3 FAs is low [134]; hence, supplemental intake may be recommended. Dietary sources of DHA are limited, with cold-water algae being the primary producers of DHA and EPA. Fish are also rich sources of DHA and EPA due to a diet consisting of algae [117]. Dietary intake of the essential fatty acid and precursor to DHA, α-linolenic acid (ALA), is generally much higher [134]. Although ALA can be metabolically converted to DHA, the conversion rate is low [135, 136]. Further, diets higher in ALA seem to limit the conversion rate by increasing the rate of ALA oxidation [137]. A dose-dependent relationship exists whereby plasma phospholipid DHA concentrations increase up to a dosage of ~ 2 g/day after which any further increase in dose negligibly increases plasma phospholipid concentration [117]. Plasma phospholipid DHA content correlates with DHA status and has been demonstrated to be a useful biomarker of DHA status in adults [138]. The neuroprotective effects of supplemental DHA observed in rodent models of TBI demonstrate the greatest efficacy when administered at a dose corresponding to 40 mg/kg/day [126], which corresponds to a dosage of approximate 3.6 g in a 90-kg athlete. Physical activity, particularly that which is long in duration and high in intensity, is known to affect plasma phospholipid composition [139], another important consideration for athletes. To determine if athletes performing heavy physical activity require higher doses, our research group recently examined the dose–response effect of supplemental DHA in American Football athletes. The lack of an observable increase in EPA, and the fact that retroconversion of DHA to EPA is regularly observed [117, 140] in those receiving a higher dose, led us to conclude that American Football athletes may require a higher dose. However, in addition to performing heavy physical activity, American Football athletes are larger than the average population with greater increases in height, weight, and body mass index when compared with all other sports over the last several decades [141]. Thus, while the optimal dosage for athletes, particularly those of larger size (i.e., ice hockey, rugby) may be higher than that reported for the average population, athletes of smaller stature and body mass may only require 2 g/day. The side effects of ω-3 FA supplementation are largely limited to gastrointestinal symptoms and poor palatability and may include malodorous belching, nausea, diarrhea, and acid reflux [29]. While concerns of significant bleeding have been raised, there is no convincing evidence of clinically significant bleeding associated with ω-3 FA supplementation [142] and those aforementioned gastrointestinal symptoms are experienced by a small percentage of those supplementing with ω-3 FAs [29].

### Combination of DHA and Curcumin

The likely differing mechanisms by which the aforementioned nutrients and nutraceuticals provide neuroprotective effects suggest that a combination may afford greater protection. Wu et al. [93] recently reported that the combination of curcumin and DHA potentiated the effect of either alone. Rats fed curcumin plus DHA reportedly had higher levels of BDNF and reduced markers of lipid peroxidation compared with control animals or those fed DHA or curcumin alone. Further, as evidenced by enhanced stability of DHA, curcumin plus DHA effectively normalized enzymes required in the metabolism of DHA, Δ6-desaturase, and 17β-hydroxysteroid dehydrogenase (HSD). Cognitive outcomes reflected those of biological markers with the combination enhancing learning ability to a greater extent [93]. Though the exact mechanism by which the combination enhanced outcomes is unknown, the normalization of enzymes associated with DHA metabolism is likely the cause. In a more recent study, researchers from that same group demonstrated that curcumin enhanced the conversion of ALA to DHA in animals fed a diet rich in ALA in combination with curcumin [143]. Similar to that observed in their previous study [93], an increase in enzymes necessary in the metabolism of DHA, Δ6-desaturase, and elongase 2, was observed. The increase in conversion resulted in a higher content of DHA in the brain [143]. Therefore, there is precedent for the potential augmentation of neuroprotection through the combination of nutrients and nutraceuticals.

### Human Studies

Up until this section, studies that have been highlighted have involved the use of animal models, specifically rodent models, of mTBI and TBI. Despite evidence as early as the year 2000 suggesting a neuroprotective role of supplementation, no study to date has been conducted in humans. This is likely due to the difficulties of conducting well designed, large-scale clinical trials in an athletic population. Athletes participating in contact sports are at risk for sustaining sports-related concussion and also show evidence of neurological damage in the absence of a concussion diagnosis. Thus, contact sport athletes represent a unique population in which to examine the potential neuroprotective effects of a nutritional intervention.

To that end, our research group recently published results from a study examining the effects of DHA supplementation on a biomarker of head trauma in American Football athletes [29]. These athletes are routinely exposed to head impacts that vary in magnitude and number over the course of the season [144, 145], resulting in some level of damage as documented via advanced neuroimaging techniques [20, 23, 24, 31, 146] and blood biomarkers [28]. American Football is associated with the highest incidence of concussion [15]. Based on our data, we concluded that DHA attenuated damage as measured by serum neurofilament light (Nf-L), the most sensitive and specific marker in regard to detecting neuroaxonal injury in concussion [147], irrespective of dose [29]. However, inference from those data was limited due to a number of constraints. In an attempt to identify an optimal dosage, as highlighted in a previous section (Sect. 3.3), athletes were randomly assigned to three different treatment groups. That reduced the number of athletes in each treatment group, which was further reduced when athletes were separated by number of repetitions performed during competition. Indeed, further examination of those data suggested that the low-dose treatment group actually experienced the greatest attenuation in head trauma. There is no doubt that large-scale clinical trials are necessary to fully elucidate the potential neuroprotective effect in athletes.

## Summary and Conclusions

Contact sport athletes are routinely exposed to impacts that may result in a sports-related concussion. However, even in the absence of a concussion, it is now well known that sub-concussive impacts cause some level of detectable damage and the combination of repetitive concussive and sub-concussive impacts has the ability to cause long-term complications. While helmets are used in some contact sports, such equipment may not prevent and/or reduce the damage resulting from head trauma. While much of the focus has been on the treatment of sports-related concussion, there exists a growing interest in protection before impact. Nutritional supplementation has emerged as a potential strategy to prevent and/or reduce the deleterious effects of sports-related concussion and sub-concussive impacts. In contrast to pharmaceutical treatment, nutrients (creatine and omega-3 FAs) and nutraceuticals (curcumin) have the potential to act on multiple mechanisms within the complex neurochemical and neurometabolic sequelae that occur subsequent to concussive and sub-concussive impacts. Despite abundant evidence in rodent models as well as in the treatment of TBI in humans [148–151], the use of nutritional supplementation is not widely accepted or recommended at present. Therefore, additional research is necessary to determine the safety and efficacy of the use of nutritional interventions.
